# The Chancellor of the Exchequer and County and Private Lunatic Asylums

**Published:** 1851-04-01

**Authors:** 


### THE CHANCELLOR OF THE EXCHEQUER AND COUNTY AND PRIVATE LUNATIC ASYLUMS.

We do not conceive that it falls within our province to record political events; but such as bear upon that department of the profession to which this journal is dedicated ought not to be passed over unnoticed. While we yet write, the ministerial crisis is scarcely over; the Chancellor of the Exchequer has again to produce his budget; and we are therefore called upon to remark that some of his financial projects will, if again brought forward and adopted, very materially affect the interests of private lunatic asylums. We believe the window tax presses grievously upon all classes of the community, but it must obviously fall more onerously upon the proprietors of lunatic asylums than upon any other class, because they are under the necessity of maintaining, at a very high rental, establishments which must be plentifully provided with windows. The proposal made by the Chancellor of the Exchequer in his late financial statement is, to abolish the window duty upon all houses under the value of £20 a year; whereby as many as 120,000 houses occupied by the labouring classes will be exempted altogether from this tax. All houses above the value of £20 a year are, as a substitute for the window duty, to have a house tax levied upon them equivalent to two-thirds of the amount now paid for window tax. The Chancellor, however, proposes that houses, a portion of which may be used for exposing goods for sale, public-houses, farm-houses, &c., on account of their being establishments for carrying on business, shall have the advantage of being taxed only ninepence in the pound. We by no means complain of such houses enjoying this exemption as far as it goes, but why did not the Chancellor of the Exchequer extend the same indulgence to lunatic asylums, which are avowedly places of professional business? The new house tax, levied according to the amount of the value or rental of these establishments, will fall upon them very heavily. The argument that the value of a house or its rental is a fair test of the means of the owner or occupier, is by no means valid as respects this class of houses, the rental of which is necessarily, on account of the extent of premises and grounds required, exceedingly high. What possible criterion is it of the net income of the proprietor? The Chancellor of the Exchequer proceeded to say—"The House is aware that in some counties of England large lunatic asylums have been built, and that considerable expense has been incurred for this purpose. I think, therefore, that such counties are more entitled to our consideration than those which have not incurred such expenditure. I propose, then, to take a portion of the charge of the maintenance of pauper lunatics, *and to take a larger share of the maintenance of those confined in county asylums than of those who are confined in private establishments.* I propose to take such a portion of the expense as will leave the cost still to be borne by the parishes little more than that of maintaining ordinary paupers. It is a reason for taking some portion of this charge, that no foresight, no sacrifice, no care on the part of the ratepayers can prevent the charge from being thrown upon the parishes. It is attributed to no neglect of theirs; it is the act of Providence; and I think it is desirable to encourage them to send unfortunate individuals to the asylums. On the other hand, I think it very desirable not to encourage them to keep in the asylums those who may safely be removed, because it is notorious that the probability of a cure in such cases depends almost entirely upon parties being sent to the asylum at the earliest possible stage of the disease. I am acquainted with a case where there was great difficulty in clearing from an asylum a number of harmless idiots, in order to make room for those whose admission at an earlier period would, in all probability, have led to their speedy cure. I have not been able to make any precise inquiries on this subject, because I did not, of course, wish to intimate to any persons the course I intended to take; but I estimated the charge for this purpose at £150,000. I am speaking of the united kingdom. I propose to take a portion of the expense of all pauper lunatics confined in public asylums or private madhouses in England, Scotland, or Ireland, upon the public funds." This sum of £150,000 it is proposed to charge upon the Consolidated Fund. But why, we would fain ask, should so invidious a distinction be drawn between public and private lunatic asylums? Upon this subject a circular has been addressed to the proprietors of private lunatic asylums, by Mr. Coode, the owner of Haydock Lodge, which establishment, at the commencement of the present year, appears by the commissioners' last report, to have contained 45 private and

355 pauper patients, making a total of 400. "The Chancellor of the Exchequer," observes Mr. Coode, "in his explanation of his intentions, proposes to take two steps very decidedly unfair and injurious to the proprietors of private asylums.

"Firstly, as regards the window tax (from the mischievous action of which we have at all times been great sufferers, on account of the large number of chargeable apertures which our establishments require), he proposes, in abolishing it, to replace it by a house tax, which, on houses above £40 a year, already built, shall be two-thirds of the amount of the previous window tax; thus perpetuating to the extent of two-thirds the injustice and grievance of the repealed tax. It is clearly the interest of most of us, as it is also but plain justice, that we should not be assessed to the house tax at a higher rate than occupiers of new houses are to be—namely, at five per cent. on our rental. In my own case, the latter tax would not be one-third of what the Chancellor of the Exchequer proposes. Indeed, it is not clear why we are not as fairly entitled as the occupiers of farm-houses, beer-shops, and other shops, to be taxed at the lower rate of ninepence in the pound of our rental, our premises being equally places of business, and their extent and rental no indication of our personal means or expenditure. In opposing the meditated injustice, we shall, as soon as we have spoken out, have the aid of numerous other interests, especially of schools and boarding-houses, similarly affected.

"Secondly, he proposes to pay out of the Consolidated Fund a part, equal to nearly £10 a year to each patient, of the expenses of maintaining lunatics in asylums. But he proposes to pay more towards the maintenance of those in county than of those in private asylums. This is evidently to afford an artificial and factitious inducement to counties—apparently to be continued in perpetuity at the expense of the nation—to provide county asylums. It is a bounty, partly to be paid at our cost, to counties, to do that which their own simple interests should alone determine. It is manifestly to place us at a disadvantage in comparison with county asylums, and to make it impossible for us to do as well for our patients as they are to be enabled to do at the public cost.

"We have originated and carried on all the improvements in the treatment of the insane, which the counties only have copied, and generally not copied successfully. We have had but little encouragement, and we require no bounties from the public, to induce us to proceed in this course; but it is not endurable that the public purse, contributed to by ourselves, should be used to impede and crush us. It is for our interest to display the unfairness of this proceeding, and to show (what we have hitherto too much neglected) our, at least, equal merits with county asylums, and that our personal interests and responsibilities for the proper treatment of the insane, and the good order of our establishments, are incomparably stronger and more cogent, and in practice more operative, than those of any persons concerned in the management of county asylums: and in fine, that we deserve better treatment than we have at the hands of the government, and at all events have a claim to fair play.

"I beg to suggest to all proprietors of private houses, that we should request the Chancellor of the Exchequer to receive a deputation from us, to represent our claims and our grievances under his proposed measure. If this be not at once successful, I suggest that we should frame petitions to the House of Commons on the subject, setting forth the matter clearly and truly. This will involve little cost in comparison with the value of the object, though it is too much for one to incur for a benefit to be common to all. If all contribute, a few shillings from each will cover it. I am desirous of learning from you whether you are willing to take a part, and if so, what part, in these proceedings; whether you can supply any useful information, either as to county or private asylums; whether you will join in the deputation, or can influence any members of the House of Commons; whether you will contribute to our expenses; and if so, whether equally with all the rest, or to a limited amount. For my part, I am ready to contribute my share to the expense, and to give any assistance which my legal education, or my training to public business, or my personal influence may make available for the common object."

The unsettled state of the Ministry, and the uncertainty which appears at present to throw a cloud over all their measures, has doubtlessly prevented the proprietors of private lunatic asylums responding as they would otherwise have done to this appeal. The claims which private asylums have upon the government are entitled to the highest consideration, for, until county asylums were built (and many are only now in progress) the public was indebted entirely to the enterprise of private individuals for the protection afforded to the lunatic poor of the kingdom, who would otherwise have remained even to the present day pining in workhouses and gaols. Union is strength, and if so manifest an injustice be inflicted upon them, the proprietors of private asylums will do well to adopt Mr. Coode's suggestions. We shall, however, pause until we know definitely the course of legislation which may be proposed, and shall then return to the subject. In the meantime, we advise the proprietors of private lunatic asylums to be on the alert.

